# Tissue Damage Caused by Myeloablative, but Not Non-Myeloablative, Conditioning before Allogeneic Stem Cell Transplantation Results in Dermal Macrophage Recruitment without Active T-Cell Interaction

**DOI:** 10.3389/fimmu.2018.00331

**Published:** 2018-02-27

**Authors:** Peter van Balen, Boris van der Zouwen, Alwine B. Kruisselbrink, Matthijs Eefting, Karoly Szuhai, Ekaterina S. Jordanova, J. H. F. Falkenburg, Inge Jedema

**Affiliations:** ^1^Department of Hematology, Leiden University Medical Center, Leiden, Netherlands; ^2^Department of Pathology, Leiden University Medical Center, Leiden, Netherlands; ^3^Department of Molecular Cell Biology, Leiden University Medical Center, Leiden, Netherlands; ^4^Center for Gynaecologic Oncology Amsterdam, VUmc, Amsterdam, Netherlands

**Keywords:** HLA class II, tissue damage, allogeneic stem cell transplantation, graft-versus-host disease, macrophages, skin

## Abstract

**Introduction:**

Conditioning regimens preceding allogeneic stem cell transplantation (alloSCT) can cause tissue damage and acceleration of the development of graft-versus-host disease (GVHD). T-cell-depleted alloSCT with postponed donor lymphocyte infusion (DLI) may reduce GVHD, because tissue injury can be restored at the time of DLI. In this study, we investigated the presence of tissue injury and inflammation in skin during the period of hematologic recovery and immune reconstitution after alloSCT.

**Methods:**

Skin biopsies were immunohistochemically stained for HLA class II, CD1a, CD11c, CD40, CD54, CD68, CD86, CD206, CD3, and CD8. HLA class II-expressing cells were characterized as activated T-cells, antigen-presenting cells (APCs), or tissue repairing macrophages. In sex-mismatched patient and donor couples, origin of cells was determined by multiplex analysis combining XY-FISH and fluorescent immunohistochemistry.

**Results:**

No inflammatory environment due to pretransplant conditioning was detected at the time of alloSCT, irrespective of the conditioning regimen. An increase in HLA class II-positive macrophages and CD3 T-cells was observed 12–24 weeks after myeloablative alloSCT, but these macrophages did not show signs of interaction with the co-localized T-cells. In contrast, during GVHD, an increase in HLA class II-expressing cells coinciding with T-cell interaction was observed, resulting in an overt inflammatory reaction with the presence of activated APC, activated donor T-cells, and localized upregulation of HLA class II expression on epidermal cells. In the absence of GVHD, patient derived macrophages were gradually replaced by donor-derived macrophages although patient-derived macrophages were detectable even 24 weeks after alloSCT.

**Conclusion:**

Conditioning regimens cause tissue damage in the skin, but this does not result in a local increase of activated APC. In contrast to the inflamed situation in GVHD, when interaction takes place between activated APC and donor T-cells, the tissue damage caused by myeloablative alloSCT results in dermal recruitment of HLA class II-positive tissue repairing macrophages co-existing with increased numbers of patient- and donor-derived T-cells, but without signs of specific interaction and initiation of an immune response. Thus, the local skin damage caused by the conditioning regimen appears to be insufficient as single factor to provoke GVHD induction.

## Introduction

After allogeneic stem cell transplantation (alloSCT), severe graft-versus-host disease (GVHD) can be induced by donor T-cells recognizing normal tissues of the recipient. T-cell depletion (TCD) of the stem cell graft is an effective strategy to prevent GVHD, and alloSCT regimens using the CD52 antibody alemtuzumab for TCD or using infusion of purified CD34 cells demonstrate efficient engraftment and reduced acute GVHD (1–4). Since graft-versus-tumor reactivity is reduced after TCD alloSCT, postponed donor lymphocyte infusions (DLIs) are frequently administered after transplantation to promote antitumor reactivity with a lower risk of GVHD (2, 5–7). Reduced incidence of GVHD after TCD alloSCT with postponed DLI may partly be explained by gradual repair in time of the tissue damage caused by the pretransplant conditioning regimen. Tissue damage can accelerate the development of acute GVHD by causing release of pro-inflammatory cytokines such as TNF-α, IL-1β, and IL-6. These danger signals can activate host or donor antigen-presenting cells (APCs) that in turn present alloantigens to donor T-cells, which can activate and amplify alloreactive donor T-cell responses (8). Although tissue damage may be restored at the time of postponed DLI, GVHD after DLI applied 3–6 months after alloSCT is not uncommon (9). Furthermore, although CD4 T-cells recognize peptides presented in HLA class II molecules and HLA class II is only expressed on non-hematopoietic cells under inflammatory conditions, infusion of purified donor CD4 T-cells 3 months after TCD alloSCT with an HLA 10/10 matched, but HLA-DP mismatched donor can also result in severe GVHD caused by CD4 T-cells directed against the mismatched HLA-DP molecule from the patient (10). These findings suggest that, as a result of tissue damage after the conditioning regimen, even several months after alloSCT, activated APCs of patient origin may still be present in target tissues like skin, which may result in the initiation of an alloreactive T-cell response causing GVHD.

In this study, we investigated the hypothesis that activated APCs are present for several months in the skin due to tissue damage caused by the conditioning regimen given before alloSCT. Since beside the conditioning regimen, T-cells administered at the time of alloSCT can contribute to inflammation with the presence of activated APC due to GVHD or immune responses against pathogens, it is not possible to investigate this hypothesis after T-cell replete alloSCT. Therefore, we investigated the presence of activated APC in the skin in the absence of an alloimmune response at several time points after TCD alloSCT. We illustrate that there is no increase in HLA class II-positive cells or T-cell influx at the time of alloSCT, regardless of the type of conditioning regimen. Total body irradiation (TBI) containing myeloablative (MA) conditioning, but not non-myeloablative (NMA) conditioning or conditioning preceding autologous stem cell transplantation (autoSCT), resulted in dermal recruitment of HLA class II-positive tissue repairing macrophages, most prominent in the period of immune reconstitution 12–24 weeks after alloSCT with co-localization of T-cells, but without inflammatory interaction. In the absence of an alloimmune response, as expected, the percentage of HLA class II-positive dermal macrophages of recipient origin decreased gradually over time after alloSCT, while percentage of HLA class II-positive dermal macrophages from donor origin increased.

## Materials and Methods

### Patients

After approval by the LUMC Institutional Review Board and written informed consent from the patient according to the Declaration of Helsinki, a 4-mm punch biopsy from normal skin over the posterior iliac crest was taken from patients at the day of alloSCT or at 3, 6, 12, or 24 weeks after alloSCT in the absence of GVHD. In case of the presence of GVHD after alloSCT, a skin biopsy was taken from affected skin. Diagnosis of skin GVHD was confirmed by an experienced pathologist.

The NMA conditioning regimen preceding HLA identical alloSCT consisted of fludarabine (50 mg/m^2^ orally from days −10 to −5), busulfan (0.8 mg/kg iv four times a day on days −7 and −6), and alemtuzumab (15 mg iv on days −4 and −3). In case of a matched unrelated donor (MUD), patients were also treated with antithymocyte globulin (thymoglobulin, 1 mg/kg on day −2). MA conditioning consisted of cyclophosphamide (60 mg/kg iv on days −6 and −5) and TBI (9 Gy on day −1) in case of an HLA identical sibling donor. In case of an MUD, alemtuzumab (15 mg on days −5 and −6) and cyclosporine A (starting 1.5 mg/kg twice a day with tapering to 0 in 3 months) was given as GVHD prophylaxis. Stem cell grafts were T-cell depleted by addition of 20 mg alemtuzumab to the bag before administration (2, 11).

Skin biopsies were taken from patients at different time points after autoSCT, from healthy donors, and from affected skin of patients suffering dermatitis medicamentosa and were used as controls for the effect of chemotherapeutic conditioning in the absence of a potential allogeneic immune response, the normal situation, or an inflamed environment, respectively. The auto-SCT conditioning regimen consisted of high-dose melphalan 100 mg/m^2^ for 2 days for patients with multiple myeloma and BEAM (BCNU 300 mg/m^2^, cytosine arabinoside 100 mg/m^2^ twice daily for 4 days, etoposide 100 mg/m^2^ twice daily for 4 days, and melphalan 140 mg/m^2^) for patients with non-Hodgkin lymphoma. Waste material after cosmetic surgery was used for biopsies from healthy controls.

All skin biopsy samples were sharp dissected and snap-frozen on Tissue-Tek (Sakura Finetek, Alphen a/d Rijn, Netherlands) in cooled iso-pentane (Sigma Aldrich, Zwijndrecht, Netherlands) and cryopreserved at −80°C. Tissues obtained from controls and patients with GVHD were also fixed in 10% v/v formalin and embedded in paraffin. Total numbers of biopsy samples included in the study as well as basic patient characteristics are listed in Table S1 in Supplementary Material.

### Immunofluorescence Labeling of Skin Biopsies

To investigate the pro-inflammatory environment in the skin at various time points after transplantation, 4-µm cryopreserved sections were cut, dried for 1 h at 60°C followed by 30 min at room temperature, and fixated in acetone for 10 min. Immunohistochemical staining was performed overnight at room temperature using monoclonal antibodies (MoAbs) against CD3 (IgG2a, Ps1-ab699, Abcam, Cambridge, MA, USA) and CD8 (IgG2b, 4B11, Leica Microsystems, Rijswijk, Netherlands) as markers for T-cell infiltration in the skin and against HLA class II (IgG1, p104630, Dako, Heverlee, Belgium), HLA-DR (IgG2b, B8.11.2), and CD54 (IgG1, ab20, Abcam) as markers for activated cells. To investigate whether HLA class II-positive cells in skin biopsies were resident dermal dendritic cells (DCs), inflammatory dermal DC, or dermal macrophages, staining with MoAb against CD1c (IgG1, MCA694, Bio-Rad Laboratories, Veenendaal, Netherlands), CD11c (IgG1, B-Ly6, BD, Breda, Netherlands), CD40 (IgG1, 5C3, BD), CD54 (IgG1, ab20, Abcam), CD86 (IgG1, ab23556, Abcam), CD206 (IgG1, 19.2, BD), and CD209 (IgG1, AZM D1, BD) was performed. Afterward, slides were incubated for 1 h at room temperature with secondary fluorescently labeled goat-anti-mouse IgG2a ALEXA 546 (red), IgG2b ALEXA 647 (blue), and IgG1 ALEXA 488 (green) (Alexa, Invitrogen, Breda, Netherlands) diluted in phosphate-buffered saline with 1% bovine serum albumin. After washing, slides were covered with Mowiol 4-88 (Sigma Aldrich), and images were captured by confocal laser scanning microscope LSM510 (Zeiss, Sliedrecht, Netherlands).

To investigate whether cells were of patient or donor origin, XY-FISH was combined with surface immunofluorescent staining for biopsies taken from patients who had undergone a sex-mismatched transplantation. Directly after primary surface staining with CD3 or HLA-DR antibodies and secondary staining with the ALEXA antibodies, slides were digested for 3 min at 37°C with a pepsin solution (Dako; Code K5799) washed with PBS and postfixed with PBS containing 1% formaldehyde. After washing in PBS and dehydration in descending ethanol series, the slides were air dried. Subsequently, 10 µl probe mix containing CEP X (DXZ1) SpectrumGreen/CEP Y (DYZ3) SpectrumOrange (Abbot, Hoofddorp, Netherlands) was pipetted onto the slides, and denaturation was performed for 3 min at 80°C. Hybridization was performed overnight in a humid chamber at 37°C and after two washing steps (SCC/0.05% Tween and 0.4× SCC at 72°C for 3 min) and dehydration, slides were coverslipped with vectashield containing a nuclear stain (DAPI).

Images were captured with a confocal laser scanning microscope (LSM700, Zeiss) in a multitrack setting and analyzed with the LSM700 software.

### Statistical Analysis

Quantification of labeled cells in the dermis was done using Image J (National Institutes of Health, Bethesda, MD, USA) by measuring the dermal area count, which is a calculation of the percentage of the area taken by the labeled cells as a part of the total dermal area in the biopsy as described before (12). Computer-assisted calculations were done to estimate the percentage of colored area in respect to the total area of a part of the biopsy. Dermal area count of at least two fields of 521µm × 521 µm per biopsy was measured. Only labeled cells in the dermis were counted, and labeled cells in the epidermis were excluded from the calculation. Mean dermal area counts of HLA class II- and CD3-positive cells in skin biopsies were calculated at different time points and conditioning regimens after autoSCT, NMA, and MA alloSCT. Kruskal–Wallis test was performed to compare median dermal area counts, and in case the null hypothesis, being no differences in medians between different groups, could be rejected, *post hoc* Mann–Whitney *U*-test was performed to compare dermal area counts between two specific groups and significance was accepted as *p* < 0.05.

## Results

### No Overt Inflammatory Environment in the Skin at Time of alloSCT

**Figure 1:**
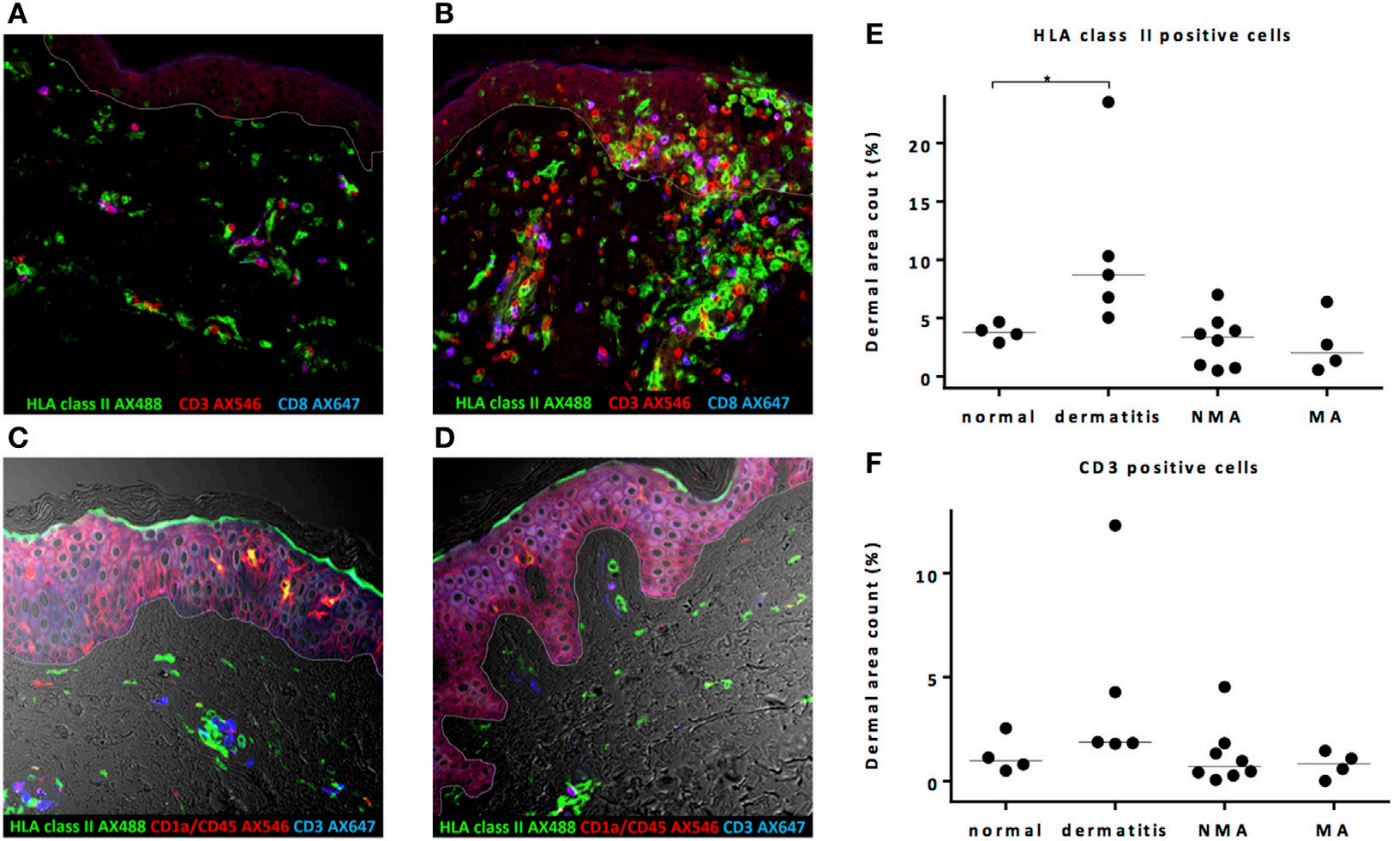
Pretransplant conditioning did not result in a pro-inflammatory environment in the skin at the time of transplantation. **(A)** In normal skin, HLA class II-expressing cells and also T-cells are present scattered over the whole dermal region. In the epidermal layer, HLA class II-expressing cells were observed sporadically. **(B)** In skin affected by dermatitis medicamentosa, dermal area count of HLA class II-expressing cells and T-cells is higher compared to normal skin. After non-myeloablative (NMA) conditioning **(C)** and myeloablative (MA) conditioning **(D)**, the presence of HLA class II-expressing cells and T-cells at the time of allogeneic stem cell transplantation (alloSCT) resembled the situation in normal skin. Dermal area counts with median were calculated for HLA class II-expressing cells **(E)** and CD3 T-cells **(F)** in skin biopsies of normal skin, skin at the time of alloSCT after NMA and MA conditioning, and skin affected by dermatitis medicamentosa. A significant difference was detected regarding HLA class II-expressing cells between normal skin and skin affected by dermatitis medicamentosa (Kruskal–Wallis test *p* = 0.019, *post hoc* Mann–Whitney *U*-test p = 0.016). White line demarks the border between dermis and epidermis.

To investigate whether conditioning before alloSCT results in an inflammatory environment in the skin coinciding with increased expression of HLA class II molecules and attraction of T-cells, the quantitative presence of HLA class II- or CD3-expressing cells in the dermal layer was substantiated by dermal area count calculation in skin biopsies taken from patients at the time of the transplantation following NMA or MA conditioning and compared to normal skin biopsies. As shown in a representative example in Figure 1A, in normal control skin biopsies, HLA class II-expressing cells were present scattered over the dermal region and showed mainly a clustering pattern around the vascular and follicular structures. In the epidermal layer, HLA class II-expressing cells were only sporadically observed. In control skin biopsies of inflamed skin from patients with dermatitis medicamentosa, an increase in HLA class II-expressing cells was observed, scattered over the dermal and epidermal layer (Figure 1B). Analysis of biopsies taken directly after pretransplant conditioning at the day of transplantation revealed no clear increase in the number of HLA class II-expressing cells. The scattered pattern of HLA class II-expressing cells strongly resembled the situation in normal skin, as illustrated by the depicted representative examples for each type of conditioning in Figures 1C,D. To substantiate the amount of HLA class II expression in the different biopsies, dermal area counts were calculated. A significant increase in HLA class II-expressing cells was observed in skin biopsies of dermatitis medicamentosa compared to normal skin (Kruskal–Wallis test *p* = 0.019, *post hoc* Mann–Whitney *U*-test *p* = 0.016). HLA class II-expressing cells were not increased in biopsies taken at the day of transplant after NMA or MA conditioning (Figure 1E).

In normal skin, both CD4 (defined as CD3-positive CD8-negative cells) and CD8 T-cells were found scattered over the dermis without signs of clustering or co-expression of HLA class II (Figure 1A), indicating a non-activated status of these T-cells. In case of dermatitis medicamentosa, an increase in T-cells (Kruskal–Wallis test *p* = 0.058) was observed in some, but not in all biopsies (Figures 1B,F). These T-cells were not activated as illustrated by the lack of co-expression of HLA class II molecules. No significant increase in the percentages of T-cells in the skin at the day of transplantation was observed, irrespective of the type of conditioning (Figure 1F). These data show that neither NMA nor MA conditioning regimen resulted in increased numbers of HLA class II-expressing cells or attraction of T-cells in the skin at the day of alloSCT.

### Increase of HLA Class II-Positive Cells in Skin Biopsies 12–24 Weeks after MA alloSCT

To analyze the activation status of dermal cells early after alloSCT and after hematopoietic reconstitution in the absence of an allogeneic immune response, we substantiated the amount of HLA class II-expressing cells and T-cells in skin biopsies taken during hematopoietic recovery (3 and 6 weeks after transplantation) and during the period of immune reconstitution (12 and 24 weeks after transplantation) by dermal area count calculation. Patients were selected not to have GVHD of the skin. Biopsies taken at various time points after transplantation were compared to biopsies taken at the day of transplantation (*T* = 0). Biopsies taken from patients treated with autoSCT following intensive chemotherapy conditioning served as a control to determine the possible influence of an alloreactive T-cell response.

**Figure 2:**
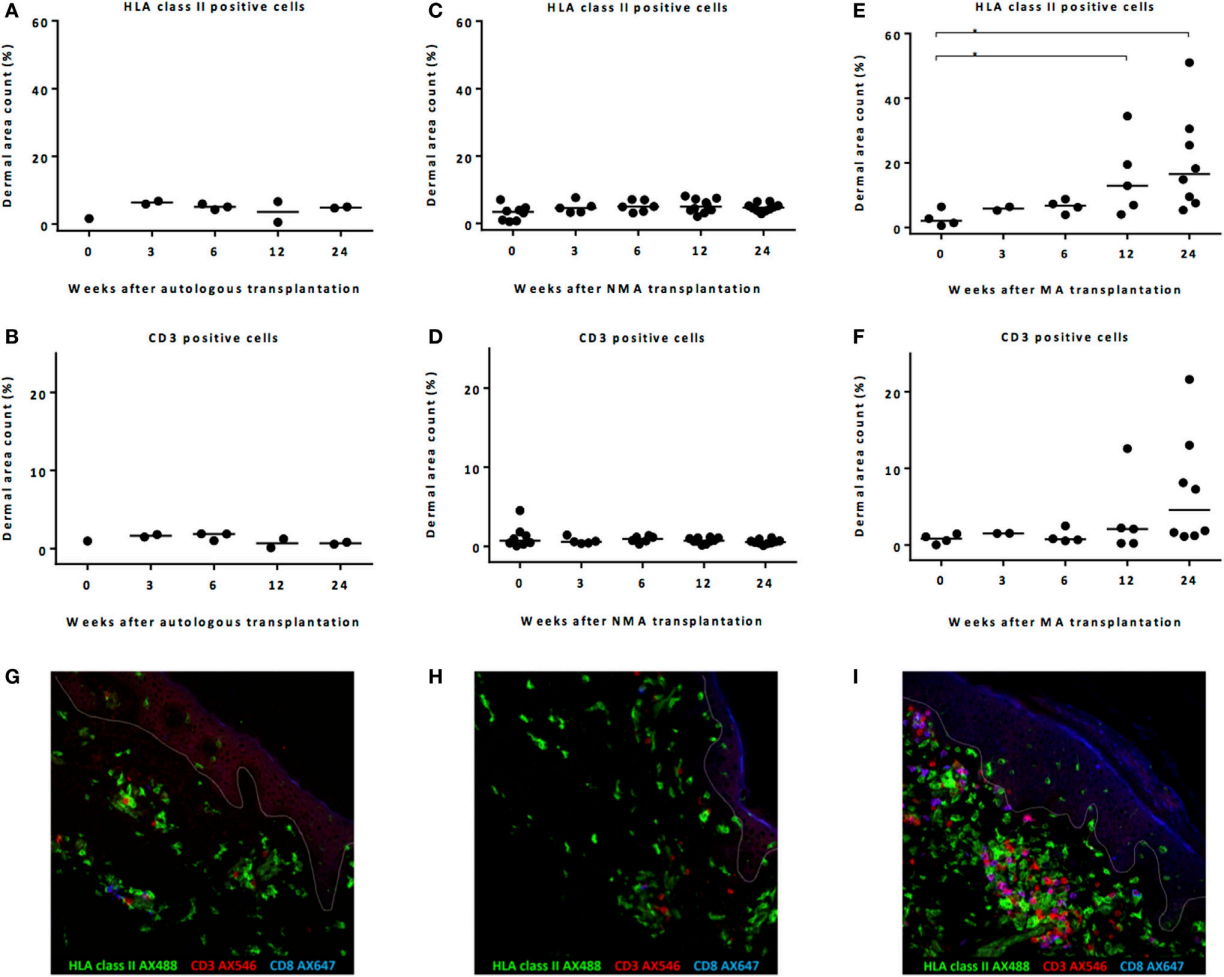
Remarkable increase in HLA class II-expressing cells after myeloablative (MA) allogeneic stem cell transplantation (alloSCT). Calculated dermal area counts with medians are depicted. No increase in HLA class II-expressing cells **(A)** or T-cells **(B)** was observed after autoSCT. Also, no increase in HLA class II-expressing cells **(C)** or T-cells **(D)** was observed after non-myeloablative (NMA) alloSCT. In contrast, following MA, alloSCT there was a significant increase in HLA class II-expressing cells after 12 and 24 weeks compared to *T* = 0 (Kruskal–Wallis test *p* = 0.020 and *post hoc* Mann–Whitney *U*-test *p* = 0.03 and 0.008) **(E)** and a non-significant increase in T-cells after 24 weeks (Kruskal–Wallis test *p* = 0.12) **(F)**. **(G–I)** Results of fluorescence microscopy of illustrative examples showing HLA class II- and CD3-expressing cells 24 weeks after autologous **(G)**, NMA **(H)**, or MA **(I)** transplantation. White line demarks the border between dermis and epidermis.

In the skin biopsies taken after autoSCT, no increase in HLA class II-expressing cells or T-cells was observed within the first 24 week after transplantation (Figures 2A,B). No increase in HLA class II-expressing cells or CD3 T-cells was found after NMA alloSCT (Figures 2C,D). In contrast, after MA alloSCT, a significant increase (*p* = 0.020, Kruskal–Wallis test) in HLA class II-expressing cells in the dermis was observed from median 2.1% of dermal region at the time of alloSCT to 12.9% at 12 weeks after alloSCT (*p* = 0.03, *post hoc* Mann–Whitney *U*-test) and to 16.5% at 24 weeks after alloSCT (*p* = 0.008, *post hoc* Mann–Whitney *U*-test) (Figure 2E). In addition, the numbers of T-cells appeared to increase within the first 24 weeks after MA alloSCT from a median of 0.83% of the dermal region at time of alloSCT to 4.6% at 24 weeks after alloSCT, although this increase did not reach statistical significance (*p* = 0.12, Kruskal–Wallis test) (Figure 2F). The immunofluorescence microscopic pictures in Figures 2G–I illustrate the presence of HLA class II-expressing cells and CD3 T-cells in biopsies taken 24 weeks after autoSCT, NMA, and MA alloSCT, respectively.

These data illustrate that after NMA alloSCT, in the absence of GVHD, no change in numbers of HLA class II-expressing cells or CD3 cells occurred. However, 12–24 weeks after MA alloSCT, despite the absence of any sign of GVHD, there was a significant increase in HLA class II-positive cells in the dermal region of the skin as well as an increase in T-cells after 24 weeks. Despite this increase, no signs of inflammation or interaction between the HLA class II-positive cells and the T-cells were detected, because T-cells appeared not to be activated illustrated by the lack of expression of HLA class II.

### Massive Inflammation in Biopsies of Acute Skin GVHD

To investigate whether the skin biopsies that showed significant increase in HLA class II-positive cells 24 weeks after MA alloSCT were immunohistochemically different from biopsies with overt inflammation, skin biopsies taken from patients suffering from acute skin GVHD within 24 weeks after alloSCT were analyzed.

**Figure 3:**
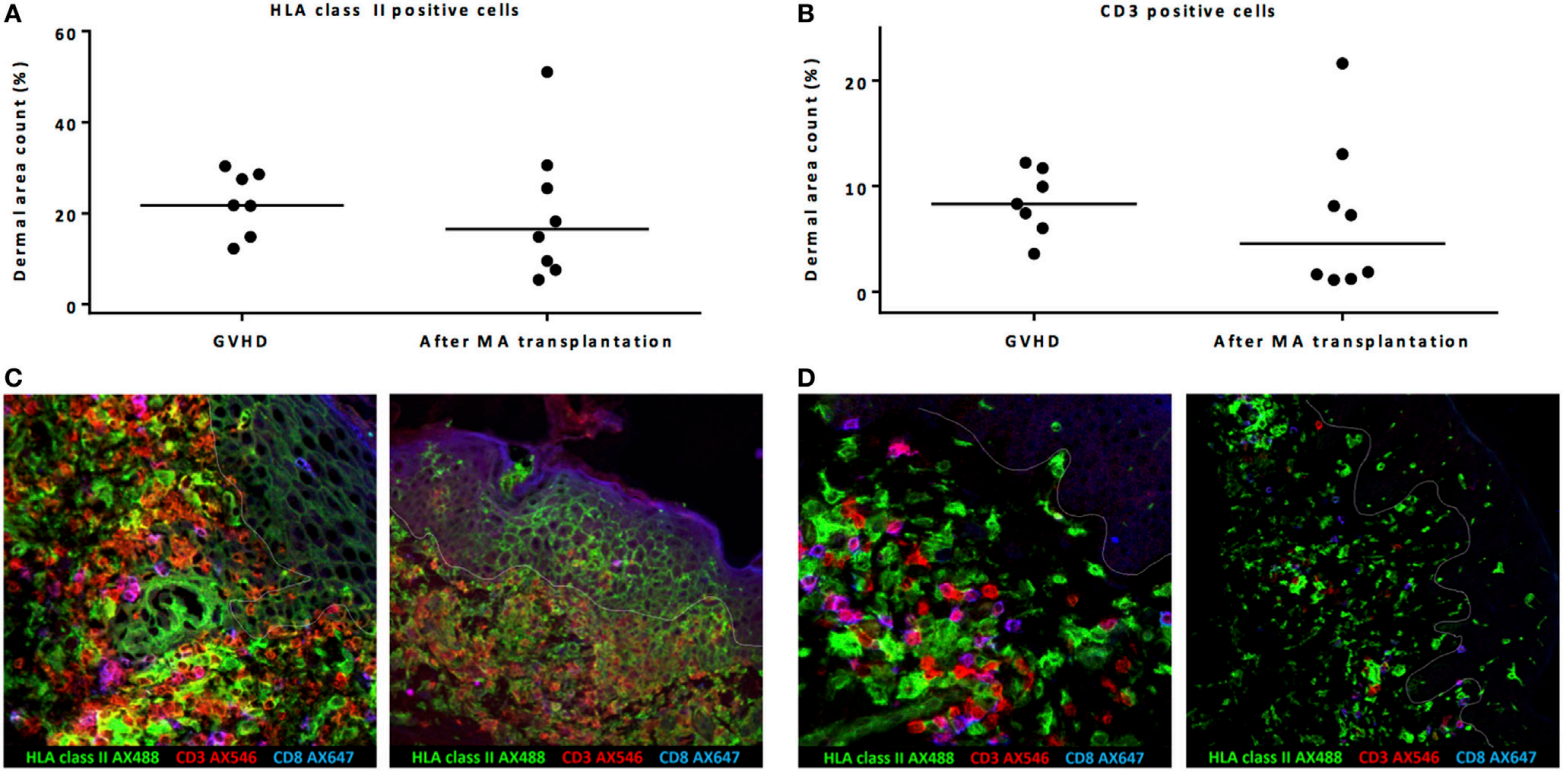
Biopsies from patients with acute skin graft-versus-host disease (GVHD) showed massive inflammation. Both dermal area count (excluding by definition the epidermal area count) of HLA class II-positive cells **(A)** and CD3-positive cells **(B)** were not significantly different between biopsies taken during acute skin GVHD and biopsies of normal skin taken 24 weeks after myeloablative (MA) allogeneic stem cell transplantation (alloSCT). However, immunofluorescence microscopy showed honeycomb pattern of HLA class II expression on epidermal cells and upregulation of HLA class II expression on T-cells (yellow hued cells) during GVHD as signs of inflammation **(C)**, while in normal skin biopsies 24 weeks after MA alloSCT, co-localization of HLA class II-positive cells and T-cells without interaction was observed **(D)**. Pink hued cells represent CD8-positive CD3 T-cells. White line demarks the border between dermis and epidermis.

Dermal area counts of HLA class II-expressing cells and CD3 T-cells were not significantly different in biopsies taken from patients during acute skin GVHD compared to biopsies taken from patients without GVHD 24 weeks after MA alloSCT (Figures 3A,B). Of note, the epidermal area was excluded in the dermal area count calculations. However, major differences were observed between biopsies taken during GVHD or 24 weeks after MA alloSCT in the absence of GVHD. First, in biopsies taken from affected skin during GVHD, localized induction of HLA class II expression in a honeycomb pattern became visible in the epidermal layer (Figure 3C), illustrating upregulation of HLA class II expression on epidermal keratinocytes as a result of inflammation. This phenomenon was not observed in skin without GVHD where the presence of HLA class II-positive cells remained limited to the dermal region (Figure 3D). Second, in GVHD biopsies, the T-cells showed overt upregulation of HLA class II expression, illustrating activation of these T-cells. Skin without GVHD showed an increase in both HLA class II-positive cells and T-cells, but without signs of interaction or upregulation of HLA class II expression on the T-cells, indicating that the HLA class II-expressing cells did not function as APCs for the T-cells present (Figures 3C,D).

These data show that in biopsies of skin affected by GVHD, massive inflammation is present, while the observed increase in HLA class II-positive cells during the immune reconstitution period after MA alloSCT is rather co-localization of HLA class II-positive cells with T-cells without signs of interaction or inflammation.

### Recruitment of Macrophages, Rather than Activated APC, Caused the Increase in HLA Class II-Expressing Cells after MA alloSCT

**Figure 4:**
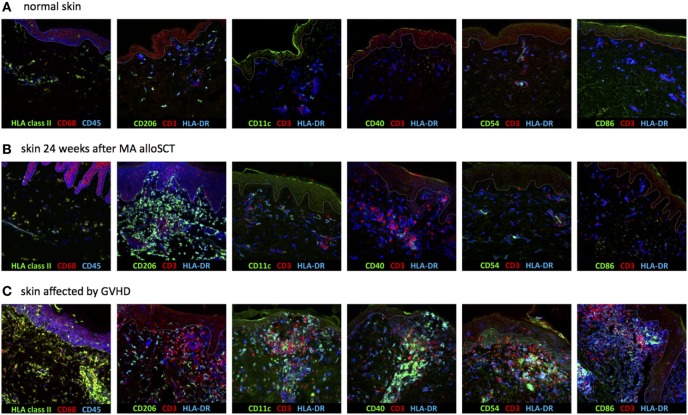
Dermal HLA class II-expressing cells seen after myeloablative (MA) allogeneic stem cell transplantation (alloSCT) were macrophages with non-professional antigen-presenting cell (APC) phenotype. **(A)** In normal skin, the majority of HLA class II-expressing cells were macrophages, as indicated by the expression of CD68 and CD206, while also some resident dermal dendritic cells (CD11c positive) were detected. The HLA class II-expressing cells were negative for CD40, CD54, and CD86. **(B)** Skin biopsies taken from patients in which an increase in HLA class II-positive cells was observed after MA alloSCT. HLA class II-expressing cells were CD68- and CD206-positive macrophages from which only a few expressed also CD11c. There was no expression of CD40, CD54, or CD86, so the HLA class II-expressing cells did not have the characteristics of activated professional APC. **(C)** Skin biopsies from skin affected by acute graft-versus-host disease (GVHD) showed increase in HLA class II-expressing cells with professional inflammatory APC phenotype (CD11c, CD40, CD54, and CD86 positive, while CD206 is negative). White line demarks the border between dermis and epidermis.

To determine the nature of the increased numbers of HLA class II-positive cells observed in the dermal region of the skin during immune reconstitution following MA transplantation, expression of CD1c, CD11c, CD40, CD54, CD86, and CD206 (mannose receptor) was measured, since these markers allow distinction of different subsets of dermal APC (Table 1) (13–16). Biopsies of normal skin (*n* = 2), skin 24 weeks after MA alloSCT (*n* = 5) and skin affected by GVHD (*n* = 4) were analyzed. The majority of HLA class II-expressing cells in normal skin was found to be macrophages, as indicated by the expression of CD68 on HLA class II-positive cells and expression of CD206 on HLA class II-positive cells, while also resident dermal DCs (CD11c and HLA class II-positive) could be detected. The HLA class II-positive cells did not show expression of costimulatory markers such as CD40, CD54, or CD86 (Figure 4A). Similar to normal skin, the majority of HLA class II-expressing cells found in skin biopsies taken after MA alloSCT in the absence of GVHD appeared to be macrophages (HLA class II-positive cells were expressing CD68-and HLA class II-positive cells were expressing CD206) (Figure 4B) that did not harbor the phenotype of resident or inflammatory dermal DCs illustrated by the absence of CD11c expression. This observation was identical for the HLA class II-positive cells found in skin of patients irrespective of the conditioning regimen and time point after transplantation. In accordance, these HLA class II-positive cells did not express costimulatory molecules CD40, CD54, or CD86 (Figure 4B).

**Table 1:** Phenotype of different dermal antigen-presenting cells.

Antigen-presenting cell	Immunophenotype
Resident dermal DC	HLA-DR^+^, CD11c^+^, CD1c^+^, CD14^−^
Inflammatory dermal DC	HLA-DR^+^, CD11c^+^, CD1c^−^, CD14^−^, CD40^+^, CD54^+^, CD86^+^
Dermal macrophages	HLA-DR^+^, CD14^+^, CD68^+^, CD206^+^, CD163^+^

*DC, dendritic cell; CD54, ICAM-1; CD206, mannose receptor*.

In contrast, in biopsies from inflamed skin taken during acute GVHD, the increase in HLA class II-expressing cells was not caused by macrophages, but by an increase in inflammatory DCs, because HLA class II-positive cells expressed CD11c, CD40, CD54, and CD86 in separate stainings (Figure 4C).

These data illustrate that during the immune reconstitution period 12–24 weeks after MA alloSCT, in the absence of acute GVHD, the observed increase in HLA class II-expressing cells in the dermis was caused by dermal macrophages with a non-professional APC phenotype. In contrast, in biopsies taken at the time of acute GVHD, inflammatory dermal DCs with professional APC phenotype were present.

### Gradual Replacement of Recipient Macrophages by Donor Macrophages

**Figure 5:**
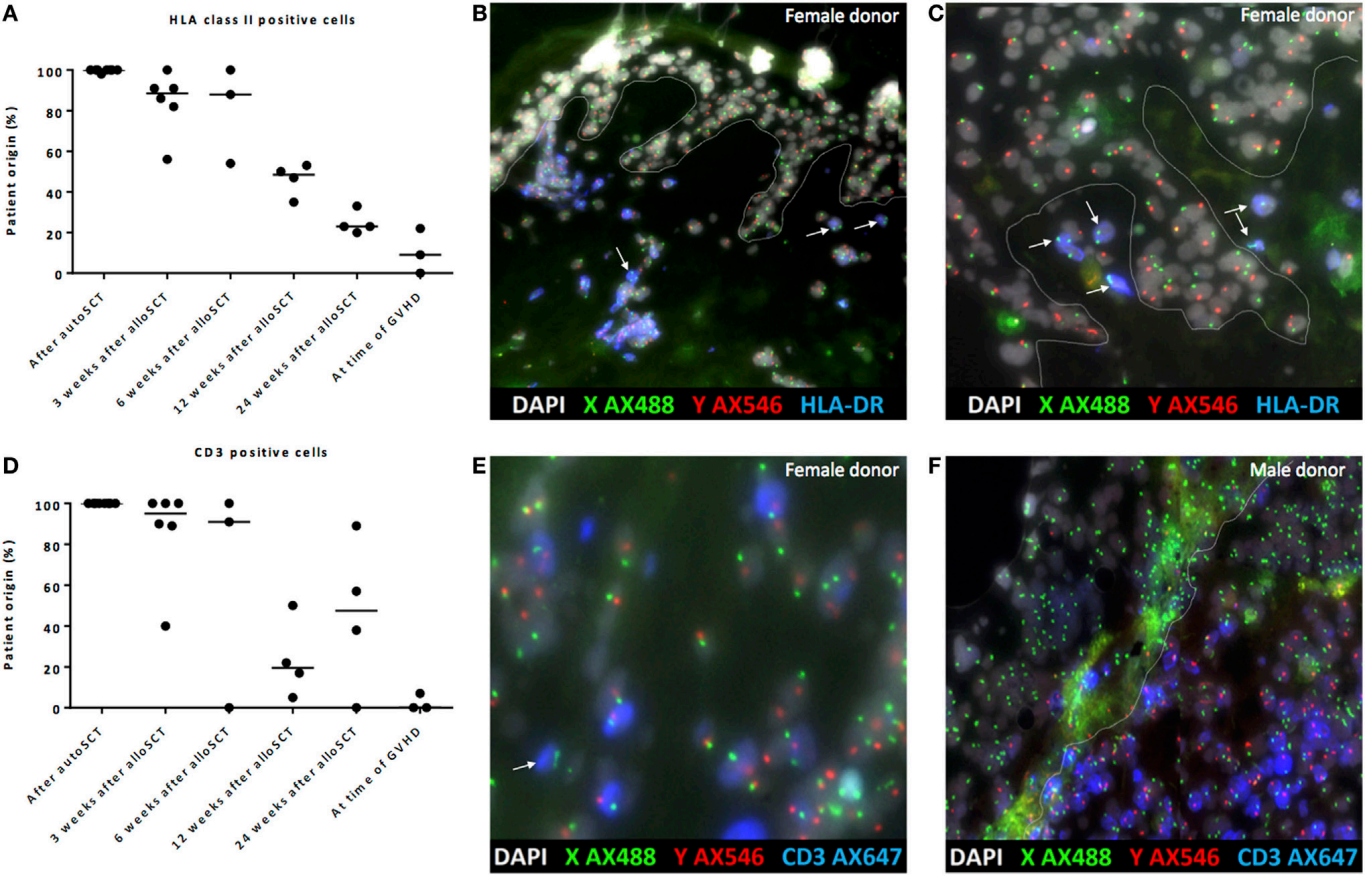
Gradual replacement of cells from recipient origin by cells of donor origin. **(A)** Percentage of recipient origin HLA class II-positive cells in the skin declined over time after allogeneic stem cell transplantation (alloSCT), but remained present even after 24 weeks. After autologous stem cell transplantation (autoSCT), no mixed chimera were observed, and in biopsies taken at the time of graft-versus-host disease (GVHD), the majority of HLA class II-positive cells were from donor origin. **(B)** Skin biopsy taken 3 weeks after alloSCT from a male patient with a female donor. All epithelial cells were XY, and nearly all HLA class II-positive cells were XY although some HLA class II-positive cells were XX (white arrows). **(C)** Skin biopsy taken 24 weeks after alloSCT from a male patient with a female donor. All epithelial cells were XY and all HLA class II-positive cells in this part of the biopsy were XX (white arrows). **(D)** Big differences were observed in the skin biopsies regarding the chimerism of CD3-positive cells. **(E)** 3 weeks after alloSCT, nearly all CD3-positive cells were from patient origin, except those indicated with a white arrow (male patient with female donor). **(F)** At the time of GVHD all CD3-positive cells were from donor origin (infiltrate of male donor CD3-positive cells in the skin of a female patient). White line demarks the border between dermis and epidermis.

To investigate whether the observed dermal macrophages with non-professional APC phenotype after alloSCT were of patient or donor origin, XY-FISH combined with staining of HLA class II was performed after alloSCT from patients with a gender-mismatched donor. Skin biopsies taken from patients after auto-SCT were used as negative controls. After alloSCT, a gradual decline of the percentages of patient-derived HLA class II-positive cells was observed, and HLA class II-positive cells of patient origin were replaced by cells of donor origin. However, even 24 weeks after alloSCT, HLA class II-positive cells of patient origin could be detected in the skin (Figures 5A–C). In the first 3–6 weeks after transplantation, in the absence of GVHD dermal T-cells were found to be predominantly of patient origin, whereas from 12 weeks after transplantation, a mixture of patient- and donor-derived T-cells was found. As expected, biopsies taken from the affected skin during active GVHD showed massive infiltration of donor T-cells (Figures 5D–F).

These data illustrate that early after transplantation, in the absence of an alloreactive immune response, the majority of HLA class II-positive cells were of patient origin, whereas late after transplantation, the majority of HLA class II-positive cells were of donor origin, illustrating the gradual replacement of patient macrophages by donor macrophages.

## Discussion

Our data illustrate that the conditioning regimen before alloSCT did not directly cause an inflammatory environment in the skin at the day of infusion of the stem cell graft. In the absence of GVHD, after hematologic recovery following TBI containing MA alloSCT, HLA class II-positive macrophages, but not cells with a phenotype of professional APC were recruited to the dermal region of the skin. These HLA class II-positive macrophages are most probably involved in repairing tissue damage. T-cells were also recruited to the dermal area, but did not show activation or interaction with the HLA class II-positive cells. In contrast, after conditioning regimens lacking TBI, no increase in dermal macrophages or T-cells was observed during the phase of hematologic and immunologic recovery. In the absence of GVHD, patient chimerism of dermal HLA class II-positive cells gradually changed over time to a median of 23% at 6 months after alloSCT, but patient-derived HLA class II-positive cells remained detectable. Skin affected by GVHD also contained high number of T-cells and HLA class II-expressing cells, but with an activated APC phenotype. In these biopsies, strong interactions between APC and T-cells were seen, coincided by the activation of T-cells and local inflammation resulting in upregulation of HLA class II molecules also on epidermal cells. Probably due to destruction of recipient-derived APC by donor T-cells during GVHD, these biopsies only contained APC of donor origin.

Although in concordance with previous studies, which illustrated HLA class II expression in skin biopsies after alloSCT in the absence of GVHD and T-cells infiltrating the skin with upregulation of HLA class II on non-hematopoietic cells occurring during GVHD (17, 18), our findings give novel insights. First, the HLA class II-positive cells detected after alloSCT in the absence of GVHD turned out not to be APC, but macrophages. High expression of CD206 on HLA class II-positive cells was found, which is predominantly present on macrophages playing a role in later stages of tissue repair in the skin and in the remodeling phase (19). Second, an increase in T-cells after MA alloSCT in the absence of GVHD was observed without inflammation or interaction with the HLA class II-positive cells. This may be explained by the presence of regulatory T-cells or γδ T-cells known to be involved in tissue repair or T-cells present for the purpose of immune surveillance (20, 21). We observed an increase in tissue repairing macrophages only after TBI containing MA and not after other conditioning regimens, indicating that TBI probably is the main contributor to the tissue damage. Although no inflammation or interaction between macrophages and T-cells was observed after MA alloSCT in the absence of GVHD, the increased presence of HLA class II-positive macrophages may result in higher susceptibility to an immune response in case subsequent DLI is administered.

After initial profound TCD, relatively high-dose DLI is required from 6 months after alloSCT to induce GVHD. In contrast, infusion of a tenfold lower dose after 3 months has already a significant risk of induction of GVHD (7, 9). It has been hypothesized that, due to tissue damage caused by the conditioning regimen, an inflammatory environment with activated APC in the skin during the hematologic and immunologic reconstitution would be the primary initiation of this response. However, our results illustrate that tissue damage is unlikely to be sufficient to create an environment for induction of an alloreactive immune response. Although tissue damage can result in the release of cytokines, this does not result in costimulatory molecule expressing activated APC able to appropriate present recipient-derived antigens to donor T-cells. This is in concordance with previous findings that, under non-inflammatory conditions, non-hematopoietic tissues are not highly susceptible to GVHD reactivity of alloreactive T-cells due to their inability to establish high avidity interactions (22).

Despite the lack of activated APC in the skin, there are several other explanations for the observed occurrence of GVHD induced by postponed application of DLI 3–6 months after TCD alloSCT. First, the alloreactive immune response can be initiated in interaction with activated APC outside the skin, followed by the release of an abundance of T-cell stimulating cytokines, upregulation of costimulatory molecules on APC and amplification of the immune response in the skin. Alternatively, an immune response to viral or bacterial infections can result in the cascade of events inducing an immune response with coinciding GVHD (10, 23, 24). Furthermore, if there is an HLA class II mismatch between patient and donor, macrophages in the skin, expressing this mismatched HLA, can be recognized by allo-HLA class II reactive donor CD4 T-cells (10, 25). Although for the initiation of an alloreactive immune response from naive donor T-cells APC are required, memory donor T-cells directed against a mismatched HLA class II molecule do not need acti-vation by APC. We previously demonstrated the occurrence of skin GVHD after the infusion of purified donor CD4 T-cells 3 months after transplantation in patients who are HLA 10/10 matched but mismatched compared to their donor regarding HLA-DP (10).

The results of our study illustrate that there is a gradual replacement of patient macrophages in the skin by donor macrophages, which is concordant with previous findings by others (26). The observation that HLA class II-positive macrophages of patient origin remain present in the skin for at least 6 months after alloSCT has to be taken into account in the risk assessment of administration of DLI post alloSCT, especially in the administration of donor T-cells in case of an HLA class II mismatch between patient and donor.

In summary, after NMA conditioning, there is no increase in HLA class II-positive cells and T-cells in the skin. TBI containing MA conditioning results in a significant increase in HLA class II-positive macrophages without APC phenotype and an increase in T-cells without activation, in the absence of interaction with each other. It is unlikely that these cells can directly initiate an alloreactive immune response in the skin. If there is an HLA class II mismatch between patient and donor, dermal macrophages may be responsible for the induction of an immune response by memory T-cells in the skin. Otherwise, initiation of an alloreactive immune response is likely to be dependent on primary events outside of the skin.

## Author Contributions

PB and BZ contributed equally as first authors. PB, BZ, AK, and ME performed research experiments and analyzed the data. PB, BZ, KS, EJ, JF, and IJ interpreted the data and wrote the manuscript.

## Conflict of Interest Statement

The authors declare that the research was conducted in the absence of any commercial or financial relationships that could be construed as a potential conflict of interest.

## References

[1] Kottaridis PD Milligan DW Chopra R Chakraverty RK Chakrabarti S Robinson S In vivo CAMPATH-1H prevents graft-versus-host disease following nonmyeloablative stem cell transplantation. Blood (2000) 96(7):2419–25. 11001893

[2] Barge RM Starrenburg CW Falkenburg JH Fibbe WE Marijt EW Willemze R Long-term follow-up of myeloablative allogeneic stem cell transplantation using Campath “in the bag” as T-cell depletion: the Leiden experience. Bone Marrow Transplant (2006) 37(12):1129–34. 10.1038/sj.bmt.1705385 16757974

[3] Pasquini MC Devine S Mendizabal A Baden LR Wingard JR Lazarus HM Comparative outcomes of donor graft CD34+ selection and immune suppressive therapy as graft-versus-host disease prophylaxis for patients with acute myeloid leukemia in complete remission undergoing HLA-matched sibling allogeneic hematopoietic cell transplantation. J Clin Oncol (2012) 30(26):3194–201. 10.1200/JCO.2012.41.7071 22869882 PMC3434978

[4] Bayraktar UD de Lima M Saliba RM Maloy M Castro-Malaspina HR Chen J Ex vivo T cell-depleted versus unmodified allografts in patients with acute myeloid leukemia in first complete remission. Biol Blood Marrow Transplant (2013) 19(6):898–903. 10.1016/j.bbmt.2013.02.018 23467126 PMC4059063

[5] Collins RH Jr Shpilberg O Drobyski WR Porter DL Giralt S Champlin R Donor leukocyte infusions in 140 patients with relapsed malignancy after allogeneic bone marrow transplantation. J Clin Oncol (1997) 15(2):433–44. 10.1200/JCO.1997.15.2.433 9053463

[6] Peggs KS Thomson K Hart DP Geary J Morris EC Yong K Dose-escalated donor lymphocyte infusions following reduced intensity transplantation: toxicity, chimerism, and disease responses. Blood (2004) 103(4):1548–56. 10.1182/blood-2003-05-1513 14576063

[7] Eefting M Halkes CJ de Wreede LC van Pelt CM Kersting S Marijt EW Myeloablative T cell-depleted alloSCT with early sequential prophylactic donor lymphocyte infusion is an efficient and safe post-remission treatment for adult ALL. Bone Marrow Transplant (2014) 49(2):287–91. 10.1038/bmt.2013.111 23933760

[8] Toubai T Mathewson ND Magenau J Reddy P. Danger signals and graft-versus-host disease: current understanding and future perspectives. Front Immunol (2016) 7:539. 10.3389/fimmu.2016.00539 27965667 PMC5126092

[9] Eefting M de Wreede LC Halkes CJ von dem Borne PA Kersting S Marijt EW Multi-state analysis illustrates treatment success after stem cell transplantation for acute myeloid leukemia followed by donor lymphocyte infusion. Haematologica (2016) 101(4):506–14. 10.3324/haematol.2015.136846 26802054 PMC5004390

[10] Stevanovic S van Bergen CA van Luxemburg-Heijs SA van der Zouwen B Jordanova ES Kruisselbrink AB HLA class II upregulation during viral infection leads to HLA-DP-directed graft-versus-host disease after CD4+ donor lymphocyte infusion. Blood (2013) 122(11):1963–73. 10.1182/blood-2012-12-470872 23777765

[11] von dem Borne PA Starrenburg CW Halkes SJ Marijt WA Fibbe WE Falkenburg JH Reduced-intensity conditioning allogeneic stem cell transplantation with donor T-cell depletion using alemtuzumab added to the graft (‘Campath in the bag’). Curr Opin Oncol (2009) 21(Suppl 1):S27–9. 10.1097/01.cco.0000357472.76337.0e 19561408

[12] Fuentes-Duculan J Suárez-Fariñas M Zaba LC Nograles KE Pierson KC Mitsui H A subpopulation of CD163-positive macrophages is classically activated in psoriasis. J Invest Dermatol (2010) 130(10):2412–22. 10.1038/jid.2010.165 20555352 PMC2939947

[13] Ochoa MT Loncaric A Krutzik SR Becker TC Modlin RL “Dermal dendritic cells” comprise two distinct populations: CD1+ dendritic cells and CD209+ macrophages. J Invest Dermatol (2008) 128(9):2225–31. 10.1038/jid.2008.56 18337829 PMC2682223

[14] Fehres CM Bruijns SC Sotthewes BN Kalay H Schaffer L Head SR Phenotypic and functional properties of human steady state CD14+ and CD1a+ antigen presenting cells and epidermal langerhans cells. PLoS One (2015) 10(11):e0143519. 10.1371/journal.pone.0143519 26605924 PMC4659545

[15] Zaba LC Krueger JG Lowes MA Resident and “inflammatory” dendritic cells in human skin. J Invest Dermatol (2009) 129(2):302–8. 10.1038/jid.2008.225 18685620 PMC2746703

[16] McLellan AD Heiser A Hart DN Induction of dendritic cell costimulator molecule expression is suppressed by T cells in the absence of antigen-specific signalling: role of cluster formation, CD40 and HLA-class II for dendritic cell activation. Immunology (1999) 98(2):171–80. 10.1046/j.1365-2567.1999.00860.x 10540215 PMC2326915

[17] Favre A Cerri A Bacigalupo A Lanino E Berti E Grossi CE. Immunohistochemical study of skin lesions in acute and chronic graft versus host disease following bone marrow transplantation. Am J Surg Pathol (1997) 21(1):23–34. 10.1097/00000478-199701000-00003 8990138

[18] Beschorner WE Farmer ER Saral R Stirling WL Santos GW. Epithelial class II antigen expression in cutaneous graft-versus-host disease. Transplantation (1987) 44(2):237–43. 10.1097/00007890-198708000-00013 3307048

[19] Novak ML Koh TJ. Macrophage phenotypes during tissue repair. J Leukoc Biol (2013) 93(6):875–81. 10.1189/jlb.1012512 23505314 PMC3656331

[20] Ali N Rosenblum MD. Regulatory T cells in skin. Immunology (2017) 152(3):372–81. 10.1111/imm.12791 28699278 PMC5629423

[21] Xu P Fu X Xiao N Guo Y Pei Q Peng Y Involvements of gamma-deltaT lymphocytes in acute and chronic skin wound repair. Inflammation (2017) 40(4):1416–27. 10.1007/s10753-017-0585-6 28540539

[22] van der Zouwen B Kruisselbrink AB Jordanova ES Rutten CE von dem Borne PA Falkenburg JH Alloreactive effector T cells require the local formation of a proinflammatory environment to allow crosstalk and high avidity interaction with nonhematopoietic tissues to induce GVHD reactivity. Biol Blood Marrow Transplant (2012) 18(9):1353–67. 10.1016/j.bbmt.2012.06.017 22796533

[23] Miller HK Braun TM Stillwell T Harris AC Choi S Connelly J Infectious risk after allogeneic hematopoietic cell transplantation complicated by acute graft-versus-host disease. Biol Blood Marrow Transplant (2017) 23(3):522–8. 10.1016/j.bbmt.2016.12.630 28017733 PMC5551893

[24] Cantoni N Hirsch HH Khanna N Gerull S Buser A Bucher C Evidence for a bidirectional relationship between cytomegalovirus replication and acute graft-versus-host disease. Biol Blood Marrow Transplant (2010) 16(9):1309–14. 10.1016/j.bbmt.2010.03.020 20353832

[25] Durakovic N Radojcic V Skarica M Bezak KB Powell JD Fuchs EJ Factors governing the activation of adoptively transferred donor T cells infused after allogeneic bone marrow transplantation in the mouse. Blood (2007) 109(10):4564–74. 10.1182/blood-2006-09-048124 17227829 PMC1885486

[26] Boeck S Hamann M Pihusch V Heller T Diem H Rolf B Kinetics of dendritic cell chimerism and T cell chimerism in allogeneic hematopoietic stem cell recipients. Bone Marrow Transplant (2006) 37(1):57–64. 10.1038/sj.bmt.1705217 16258529

