# Supplementary material for: Tissue Damage Caused by Myeloablative, but Not Non-Myeloablative, Conditioning before Allogeneic Stem Cell Transplantation Results in Dermal Macrophage Recruitment without Active T-Cell Interaction
Source: Front Immunol. 2018 Feb 27;9:331. doi: 10.3389/fimmu.2018.00331 (PMC5835032; doi:10.3389/fimmu.2018.00331)
Supplement: Supplementary file 1 [file Table_1.docx]

**Supplementary Table 1. Total numbers of skin biopsies taken under different conditions included in the study and patient characteristics.**

| Condition | Time point (weeks) | Number of biopsies | Disease of patient | Age (years) | Gender |
| --- | --- | --- | --- | --- | --- |
| Normal skin |  | 4 | n.a. | unknown | unknown |
| Dermatitis medicamentosa |  | 5 | n.a. | 42  70  41  60  52 | M  F  M  M  M |
| AutoSCT | 0  3  6  12  24 | 1  2  3  2  2 | MM  M. Hodgkin  MM  MM  M. Hodgkin  MM  DLBCL  MM  DLBCL  MM | 52  26  69  63  54  51  59  66  52  63 | F  F  F  F  M  M  M  M  M  M |
| NMA alloSCT | 0  3  6  12  24 | 8  5  6  10  10 | AML  AML  B-ALL  AML  AML  AML  MM  AML  T-PLL  Myelofibrosis  AML  MM  AML  B-ALL  AML  AML  AML  AML  MM  Follicular lymphoma  Myelofibrosis  AML  AML  Follicular lymphoma  MDS  AML  AML  Follicular lymphoma  AML  MM  CML  MM  Follicular lymphoma  MDS  Follicular lymphoma  AML  MM  T-PLL  Follicular lymphoma | 55  63  61  27  54  61  60  65  60  54  71  54  57  61  62  56  64  60  61  47  55  70  44  64  59  53  62  58  63  41  69  54  62  58  68  61  53  49  62 | F  M  M  F  M  F  M  F  F  F  F  M  F  M  F  F  M  M  M  M  M  F  F  M  F  M  M  M  M  M  M  M  M  M  F  F  M  F  F |
| MA alloSCT | 0  3  6  12  24 | 4  2  4  5  8 | CML  MDS-RAEB2  AML  AML  CML  AML  AML  B-ALL  AML  B-ALL  AML  T-ALL  AML  AML  B-ALL  AML  AML  AML  MDS-RAEB2  B-ALL  AML  MDS-RAEB2  AML | 53  37  57  41  54  38  53  21  26  29  43  50  46  53  50  50  50  39  46  47  29  56  58 | M  M  F  M  M  F  M  M  F  M  M  F  M  M  M  M  M  M  M  M  F  M  M |
| GVHD |  | 7 | T-ALL  AML  AML  M. Hodgkin  MDS-RAEB2  AML  AML | 56  25  50  38  44  46  50 | F  M  M  M  F  M  F |

AutoSCT: autologous stem cell transplantation

NMA alloSCT: allogeneic stem cell transplantation after non-myeloablative conditioning regimen

MA alloSCT: allogeneic stem cell transplantation after myeloablative conditioning regimen

GVHD: graft-versus-host disease

MM: multiple myeloma

DLBCL: diffuse large B-cell lymphoma

AML: acute myeloid leukemia

B-ALL: B-cell lymphoblastic leukemia

T-ALL: T-cell lymphoblastic leukemia

T-PLL: T-cell prolymphocytic leukemia

MDS: myelodysplastic syndrome

CML: chronic myeloid leukemia
